# Can perioperative pCO_2_ gaps predict complications in patients undergoing major elective abdominal surgery randomized to goal-directed therapy or standard care? A secondary analysis

**DOI:** 10.1007/s10877-023-01117-y

**Published:** 2024-01-22

**Authors:** Ilonka N. de Keijzer, Thomas Kaufmann, Eric E.C. de Waal, Michael Frank, Dianne de Korte-de Boer, Leonard M. Montenij, Wolfgang Buhre, Thomas W.L. Scheeren

**Affiliations:** 1 Department of Anesthesiology, University Medical Center Groningen, University of Groningen, PO Box 30.001, 9700 RB Groningen, The Netherlands; 2 Department of Anesthesiology, University Medical Center Utrecht, Utrecht, The Netherlands; 3 Department of Anesthesiology and Intensive Care, Albert Schweitzer Hospital, Dordrecht, The Netherlands; 4 Department of Anesthesiology, Maastricht University Medical Center, Maastricht, The Netherlands; 5 Department of Anesthesiology and Intensive Care, Catharina Ziekenhuis, Eindhoven, The Netherlands; 6 Edwards Lifesciences, Garching, Germany

**Keywords:** pCO_2_ gap, Hemodynamic monitoring, Postoperative Complications, Cardiac output, Goal-directed therapy

## Abstract

The difference between venous and arterial carbon dioxide pressure (pCO_2_ gap), has been used as a diagnostic and prognostic tool. We aimed to assess whether perioperative pCO_2_ gaps can predict postoperative complications. This was a secondary analysis of a multicenter RCT comparing goal-directed therapy (GDT) to standard care in which 464 patients undergoing high-risk elective abdominal surgery were included. Arterial and central venous blood samples were simultaneously obtained at four time points: after induction, at the end of surgery, at PACU/ICU admission, and PACU/ICU discharge. Complications within the first 30 days after surgery were recorded. Similar pCO_2_ gaps were found in patients with and without complications, except for the pCO_2_ gap at the end of surgery, which was higher in patients with complications (6.0 mmHg [5.0–8.0] vs. 6.0 mmHg [4.1–7.5], p = 0.005). The area under receiver operating characteristics curves for predicting complications from pCO_2_ gaps at all time points were between 0.5 and 0.6. A weak correlation between ScvO_2_ and pCO_2_ gaps was found for all timepoints (ρ was between − 0.40 and − 0.29 for all timepoints, p < 0.001). The pCO_2_ gap did not differ between GDT and standard care at any of the selected time points. In our study, pCO_2_ gap was a poor predictor of major postoperative complications at all selected time points. Our research does not support the use of pCO_2_ gap as a prognostic tool after high-risk abdominal surgery. pCO_2_ gaps were comparable between GDT and standard care. *Clinical trial registration* Netherlands Trial Registry NTR3380.

## Introduction

The pCO_2_ gap is the difference between venous and arterial carbon dioxide pressure and might be used for diagnostic, prognostic, or therapeutic purposes. First, an association between pCO_2_ gap and postoperative complications was found in patients undergoing major abdominal surgery; pCO_2_ gap was found to be higher in the proportion of patients suffering from postoperative complications [1]. In patients with septic shock, a persistently high pCO_2_ gap was associated with worse outcomes [2] and was found to be a modest predictor of mortality [3]. Second, pCO_2_ gaps reflect the adequacy of cardiac output and tissue perfusion [4]. An inverse relationship exists between cardiac output and pCO_2_ gap, and an increase in pCO_2_ gap to more than 6 mmHg is considered abnormal. Additionally, pCO_2_ gaps have been shown to reliably estimate cardiac index in a perioperative setting [5]. Third, pCO_2_ gaps may be used as a target in a goal-directed therapy (GDT) protocol. GDT uses preset hemodynamic targets to guide hemodynamic interventions, such as vasopressors, inotropes, and fluids [6]. GDT can focus on any given hemodynamic variable; however, it is recommended to use variables representing blood flow [6]. It has been shown that perioperative GDT reduces morbidity and mortality [7, 8].

The majority of studies investigating pCO_2_ gap have been conducted in critically-ill patients and the evidence for the use of pCO_2_ gaps in the perioperative setting is limited [9]. The primary aim of this study was to assess the association and predictive value of the perioperative pCO_2_ gap with a composite outcome of major postoperative complications using patient data from a randomized controlled trial comparing GDT versus standard care in patients undergoing elective high-risk abdominal surgery [10]. The secondary aim of this study was to assess the correlation between ScvO_2_ and pCO_2_ gap. Central venous oxygen saturation (ScvO_2_) is a representation of oxygen delivery and demand and can be used to assess global tissue oxygenation [11], similar to pCO_2_ gap, which reflects adequacy of cardiac output and tissue perfusion. Yet, the correlation between the two has not been assessed in patients undergoing elective high-risk abdominal surgery. Finally, pCO_2_ gaps were compared between standard care and GDT as a measure of adequacy of cardiac output, since actual cardiac output measurements were not available in this study.

## Materials & methods

**Fig. 1:**
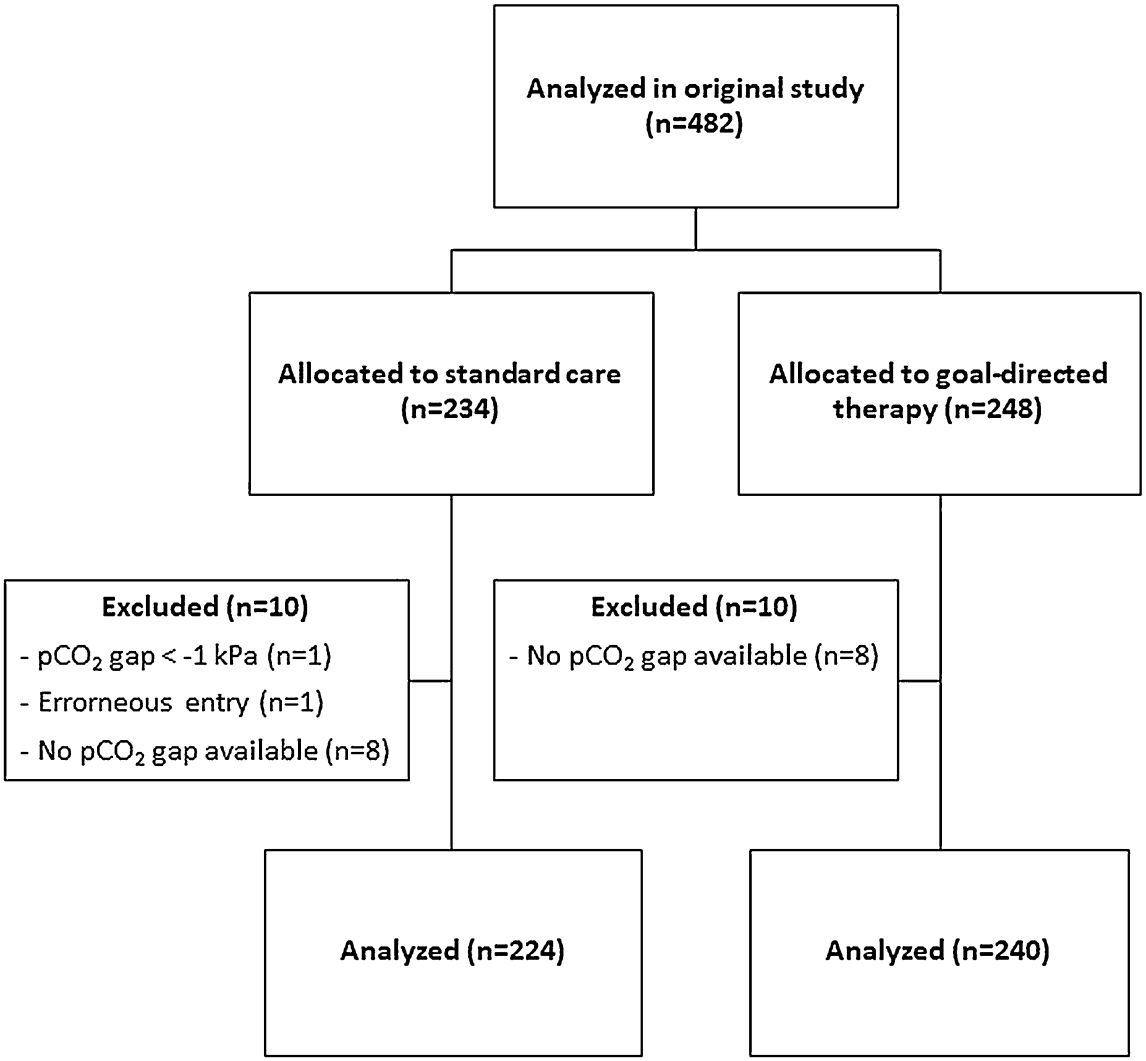
CONSORT Flow diagram

This study concerned a secondary analysis of a multicenter randomized controlled trial comparing the incidence of major complications in the first 30 days between a GDT and standard care in patients undergoing elective, high-risk abdominal surgery [10]. The study was approved by all necessary ethical review boards and written informed consent was obtained before any study procedures were conducted. This manuscript adheres to the CONSORT reporting guidelines [12]. Patients aged 18 years or older were included when they were undergoing elective, high-risk abdominal surgery. Exclusion criteria were emergency surgery, aortic valve insufficiency grade > 1, cardiac arrhythmias, contraindications to the passive leg raising test, and indication for invasive cardiac output monitoring during surgery. A CONSORT flow diagram can be found in Fig. 1.

### Study procedures

The detailed study protocol, randomization sequence, and primary study outcomes have been previously published [10, 13]. In all patients, an arterial and central venous catheter was inserted. The GDT group was additionally monitored using arterial waveform analysis (FloTrac - Vigileo, Edwards Lifesciences, Irvine, CA, USA) and treated according to a cardiac index (CI) targeted GDT algorithm [10]. The GDT algorithm was initiated after anesthesia induction and continued for a maximum of 24 h or until PACU/ICU discharge, whichever occurred first. Stopping criteria were arrhythmia, (suspected) myocardial ischemia, and cardiac decompensation.

Arterial and central venous blood samples were simultaneously obtained at four time points: after induction, at the end of surgery, at PACU/ICU admission, and at PACU/ICU discharge. All blood samples were directly analyzed per local practice.

### Outcomes

The primary outcome was to assess the association between pCO_2_ gap and a composite outcome of major complications. The composite outcome of major complications, as considered by the Accordion Severity Grading system [14], consisted of the following: death, cardiac arrest, acute myocardial infarction, acute pulmonary edema, cerebrovascular accident, prolonged mechanical ventilation, pulmonary embolism, pneumonia, respiratory insufficiency, acute kidney injury, anastomotic leakage, other gastro-intestinal complications, wound infection and severe sepsis [10]. Therefore, all subjects from the original study who did not have at least one pCO_2_ gap value were excluded from this analysis, resulting in 464 remaining subjects of the original 482 subjects. pCO_2_ gaps larger than + 30.0 mmHg or smaller than − 7.5 mmHg were considered artifacts and were removed [15].

Additionally, arterial and central venous oxygen saturation, lactate, and pH were compared between both groups. Arterial oxygen saturation measurements, which were lower than their venous counterpart, were considered artifacts, and both the venous and arterial measurements were removed (n = 2). An additional 12 venous oxygen saturation values were removed as they were in the range of 5 to 7% and were considered artifacts. For lactate, three erroneous entries were removed, one was a negative value and the other two were below the detectable limit. Subsequently, the correlation between pCO_2_ gaps and central venous oxygen saturation was assessed.

Last, the difference in perioperative pCO_2_ gaps between GDT and standard care were assessed as a measure of the adequacy of cardiac output.

### Statistical analysis

Continuous variables were presented as mean and standard deviation (SD) or median and interquartile ranges (IQR) when indicated. Normality was visually assessed using Q-Q plots. Categorical data were presented as numbers and percentages. Continuous data were compared using the Mann-Whitney-U test since none of the continuous data met the parametric assumption. Categorical data were compared using Chi-square or Fisher’s exact test when indicated. A Spearman correlation was used to assess the correlation between pCO_2_ gap and ScvO_2_. A logistic regression analysis was performed to assess the association between pCO_2_ gap and major complications. Subsequently, Receiver Operating Characteristics (ROC) curves were plotted and the areas under the ROC (AUROC) curve were assessed. Missing data were coded as missing, and no imputation was used. A p-value of 0.05 was considered statistically significant. All analyses were performed using RStudio (version 1.4.1106, RStudio, Vienna, Austria).

## Results

### Patient characteristics

A total of 464 patients were included in this secondary analysis (Fig. 1). Eighteen patients were excluded from the original analysis since pCO_2_ gaps for all four time points were missing. The age in the GDT group was 65 [59–73] years versus 67 [61–75] years in the control group. Other patient characteristics, e.g., gender, body mass index, American Society of Anesthesiology physical status, comorbidities, and type of surgery, can be found in Table 1.

**Table 1:** Patient characteristics

	GDT (N = 240)	Control (N = 224)
Age (years)	65 [59–73]	67 [61–75]
Gender, male	163 (68)	146 (65)
BMI (kg/m^2^)	25.2 [22.8–28.4]	25.8 [23.0-28.8]
*ASA*		
I	16 (7)	23 (10)
II	129 (54)	120 (54)
III	90 (38)	80 (36)
IV	4 (2)	1 (0)
*Comorbidities*		
Hypertension	106 (44)	112 (50)
Heart failure	25 (11)	29 (13)
CAD/Myocardial infarction	44 (18)	35 (16)
Diabetes Mellitus	49 (20)	52 (23)
COPD/Asthma	42 (18)	41 (18)
Restrictive lung disease	7 (3)	7 (3)
Impaired renal function	12 (5)	18 (8)
Impaired liver function	12 (5)	11 (5)
Inflammatory bowel disease	2 (1)	2 (1)
Musculoskeletal pathology	8 (3)	10 (4)
Increased bleeding tendency	77 (33)	79 (35)
*Type of surgery*		
AAA	32 (14)	34 (16)
Colorectal surgery	23 (10)	24 (11)
Resection of large soft tissue mass	12 (5)	11 (5)
Esophageal resection	41 (18)	35 (16)
Total gastrectomy	12 (5)	9 (4)
Whipple/PPPD	101 (43)	92 (42)
Other abdominal surgery	13 (6)	13 (6)

Data are presented as median [interquartile range], or numbers (%)

*GDT *goal-directed therapy, *BMI *Body Mass Index, *ASA *American Society of Anesthesiologists, *CAD *Coronary Artery Disease, *COPD *Chronic Obstructive Pulmonary Disease, *AAA *Abdominal Aorta Aneurysm, *PPPD *Pylorus Preserving Pancreaticoduodenectomy. *ICU *intensive care unit, *PACU *Post Anesthesia Care Unit

### Association between pCO_2_ gaps and major complications

**Fig. 2:**
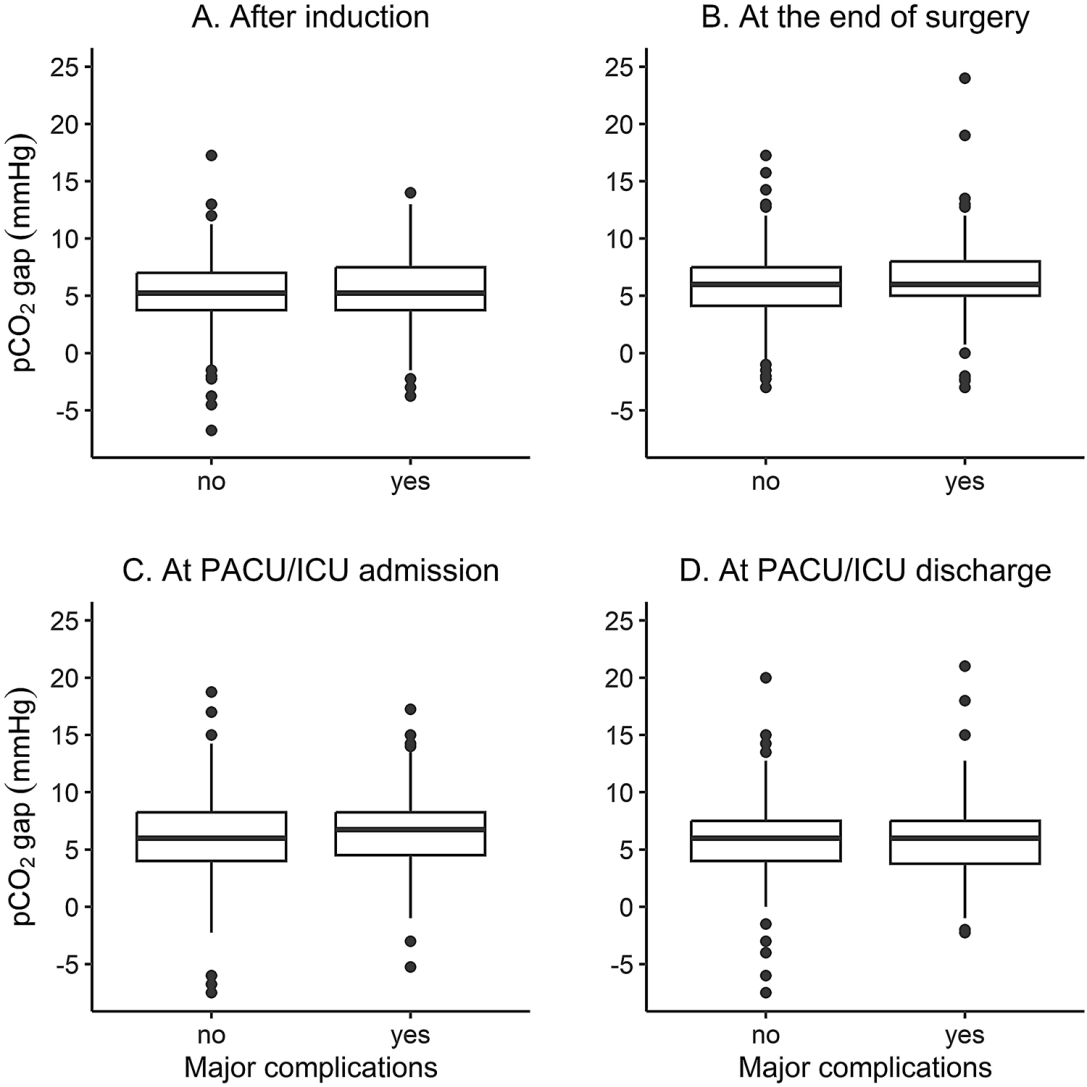
pCO_2_ gaps at different timepoints: **A** After induction, **B** At the end of surgery, **C** At PACU/ICU admission, **D** At PACU/ICU discharge. The boxes represent the 25th through 75th percentile including the median. The whiskers represent the highest or lowest values to a maximum of 1.5 times the IQR, otherwise the data point is considered an outlier (black dots)

Two hundred patients (43%) suffered from major complications. pCO_2_ gaps after induction of anesthesia, PACU/ICU admission, and PACU/ICU discharge were similar for patients with and without postoperative complications (Table 2; Fig. 2). At the end of surgery, the patients with major complications had a higher pCO_2_ gap than those without major complications (6.0 mmHg [5.0–8.0] vs. 6.0 mmHg [4.1–7.5], p = 0.005).

**Table 2:** pCO_2_ gap in patients with and without major complications

	Without major complications (N = 264)	With major complications (N = 200)	p-value
pCO_2_ gap after induction of anesthesia (mmHg)	5.3 [3.8-7.0]	5.3 [3.8–7.5]	0.762
pCO_2_ gap at the end of surgery (mmHg)	6.0 [4.1–7.5]	6.0 [5.0–8.0]	0.005*
pCO_2_ gap at ICU/PACU admission (mmHg)	6.0 [4.0-8.3]	6.8 [4.5–8.3]	0.402
pCO_2_ gap at ICU/PACU discharge (mmHg)	6.0 [4.0-7.5]	6.0 [3.8–7.5]	0.966

pCO_2_ gap in patients with and without major complications

*p < 0.05. Data are presented as median [interquartile ranges]

Logistic regression showed an association between pCO_2_ gap at the end of surgery and major complications (χ^2^(1) = 5.77, p = 0.016 (Table 3)) with an odds ratio of 1.08 (95%CI 1.01–1.16). The AUROCs for predicting postoperative complications were 0.508 (95%CI 0.454–0.563) for pCO_2_ gap after induction, 0.578 (95%CI 0.524–0.633) at the end of surgery, 0.524 (95%CI 0.468–0.580) at PACU/ICU admission, and 0.499 (95%CI 0.438–0.560) at PACU/ICU discharge. Since all AUROCs were between 0.5 and 0.6, cut-off values were not further explored.

**Table 3:** Logistic regression of pCO_2_ gap at the end of surgery for the prediction of major complications

	B (SE)	Odds ratio (95% CI)
Intercept	− 0.74 (0.23)**	
pCO_2_ gap at the end of surgery	0.08 (0.03)*	1.08 (1.01–1.16)

Logistic regression of pCO_2_ gap at the end of surgery for the prediction of major complications

Model χ^2^(1) = 5.77, p = 0.016. R^2^ = 0.01 (Hosmer-Lemeshow), R^2^ = 0.01 (Cox-Snell), R^2^ = 0.02 (Nagelkerke)

*p < 0.01, **p < 0.001

### Correlation central venous oxygen saturation and pCO_2_ gaps

**Fig. 3:**
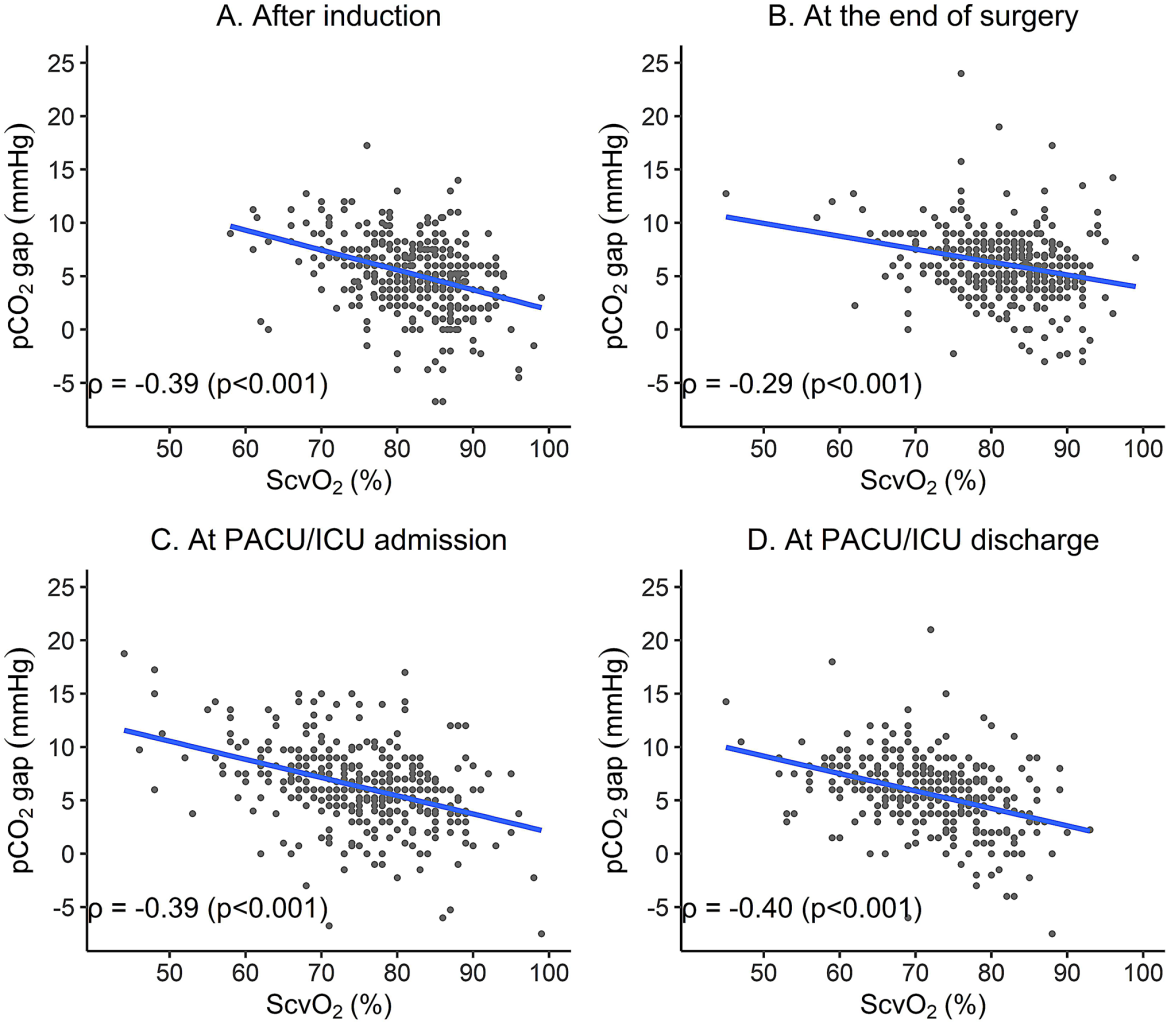
Correlation between pCO_2_ gap and ScvO_2_. pCO_2_ gap = difference between venous and arterial carbon dioxide pressure. ScvO_2_ = central venous oxygen saturation **A** Correlation between pCO_2_ gap and ScvO_2_ obtained after induction of anesthesia. **B** Correlation between pCO_2_ gap and ScvO_2_ obtained at the end of surgery. **C** Correlation between pCO_2_ gap and ScvO_2_ obtained at PACU/ICU admission. **D** Correlation between pCO_2_ gap and ScvO_2_ obtained at PACU/ICU discharge

A weak correlation was found between ScvO_2_ and pCO_2_ gaps at all four time points (after induction ρ = -0.39 (p < 0.001), at the end of surgery ρ = −0.29 (p < 0.001), at PACU/ICU admission ρ= −0.39 (p < 0.001), and at PACU/ICU discharge ρ= −0.40 (p < 0.001), Fig. 3).

### Blood gas analyses GDT vs. control

Arterial pCO_2_, venous pCO_2_, and pCO_2_ gaps at all time points did not significantly differ between GDT and standard care (Table 4). Central venous oxygen saturation after surgical closure was significantly higher in the GDT group compared to the control group (83% [79–87%] vs. 82% [77–86%], p = 0.032, Table 4). Lactate was higher in the GDT group compared to the control group after surgical closure (2.1 mmol L^−1^ [1.3–3.0 mmol L^−1^] vs. 1.8 mmol L^−1^ [1.3–2.6 mmol L^−1^], p = 0.046, Table 4). The pH was similar between the two groups at any of the time points (Table 4).

**Table 4:** Blood gas analysis between GDT group and control group

	GDT	Control	p-value
Arterial pCO_2_ after induction of anesthesia (mmHg)	40.0 [37.5–42.9]	40.0 [37.0-42.8]	0.839
Arterial pCO_2_ at the end of surgery (mmHg)	40.5 [37.5–44.3]	41.0 [38.3–44.3]	0.463
Arterial pCO_2_ at ICU/PACU admission (mmHg)	42.0 [38.3–45.0]	42.0 [38.3–46.0]	0.386
Arterial pCO_2_ at ICU/PACU discharge (mmHg)	40.5 [37.5–44.0]	40.5 [38.3–43.5]	0.442
Venous pCO_2_ after induction of anesthesia (mmHg)	45.8 [42.8–48.0]	45.0 [42.0–48.0]	0.090
Venous pCO_2_ at the end of surgery (mmHg)	47.0 [44.0-50.3]	47.0 [44.0-49.9]	0.966
Venous pCO_2_ at ICU/PACU admission (mmHg)	48.0 [45.0–51.0]	48.8 [45.0-52.5]	0.262
Venous pCO_2_ at ICU/PACU discharge (mmHg)	46.5 [43.5–49.5]	45.8 [43.5–50.1]	0.925
pCO_2_ gap after induction of anesthesia (mmHg)	6.0 [3.8–7.5]	5.3 [3.2–6.9]	0.094
pCO_2_ gap at the end of surgery (mmHg)	6.0 [5.0–8.0]	6.0 [4.0-7.5]	0.111
pCO_2_ gap at ICU/PACU admission (mmHg)	6.0 [4.5–8.3]	6.0 [4.0-8.3]	0.882
pCO_2_ gap at ICU/PACU discharge (mmHg)	6.0 [3.8–7.6]	6.0 [3.8–7.5]	0.431
Arterial O_2_ saturation after induction of anesthesia (%)	99 [98–99]	99 [98–99]	0.411
Arterial O_2_ saturation at the end of surgery (%)	99 [98–99]	99 [98–99]	0.982
Arterial O_2_ saturation at ICU/PACU admission (%)	98 [97–99]	98 [96–99]	0.062
Arterial O_2_ saturation at ICU/PACU discharge (%)	97 [95–98]	96 [95–98]	0.724
Venous O_2_ saturation after induction of anesthesia (%)	82 [77–87]	83 [78–87]	0.496
Venous O_2_ saturation at the end of surgery (%)	83 [79–87]	82 [77–86]	0.032*
Venous O_2_ saturation at ICU/PACU admission (%)	76 [71–82]	75 [69–80]	0.111
Venous O_2_ saturation at ICU/PACU discharge (%)	71 [66–77]	71 [66–78]	0.915
Arterial pH after induction of anesthesia	7.40 [7.37–7.43]	7.40 [7.36–7.43]	0.438
Arterial pH at the end of surgery	7.36 [7.31–7.39]	7.36 [7.33–7.40]	0.105
Arterial pH at ICU/PACU admission	7.35 [7.32–7.38]	7.35 [7.32–7.38]	0.529
Arterial pH at ICU/PACU discharge	7.38 [7.35–7.41]	7.38 [7.35–7.40]	0.874
Lactate after induction of anesthesia (mmol L^−1^)	1.4 [1.1–1.8]	1.4 [0.5–1.8]	0.851
Lactate at the end of surgery (mmol L^−1^)	2.1 [1.3-3.0]	1.8 [1.3–2.6]	0.046*
Lactate at ICU/PACU admission (mmol L^−1^)	1.9 [1.2–2.8]	1.7 [1.2–2.4]	0.178
Lactate at ICU/PACU discharge (mmol L^−1^)	1.4 [1.0–2.0]	1.4 [1.0–2.0]	0.834

Blood gas analysis between GDT group and control group

*GDT* goal-directed therapy, *ICU*  intensive care unit, *PACU* post anesthesia care unit

*p < 0.05. Data are presented as median [interquartile ranges]

Bold means significant, but can be omitted since there is also a *

## Discussion

This study is the largest multicenter trial to date concerning the prognostic ability of pCO_2_ gaps in a non-cardiac surgical population. We found that patients who suffered from major postoperative complications had a statistically significantly higher pCO_2_ gap at the end of surgery than patients without major complications. However, this difference cannot be considered clinically significant. Furthermore, pCO_2_ gap was a poor predictor of major postoperative complications at any of the selected time points. The GDT group did however, have higher ScvO_2_ and higher lactate at the end of surgery than the control group. Perioperative pCO_2_ gaps were similar for the GDT and control group in patients undergoing high-risk abdominal surgery.

The patients who suffered from major postoperative complications had a higher pCO_2_ gap at the end of surgery (6.0 mmHg [5.0–8.0]) compared to patients without major complications (6.0 mmHg [4.1–7.5], p = 0.005). Although the small difference in pCO_2_ gap between both groups was statistically significant, it was not considered clinically relevant and may also be explained by multiple testing. A difference in pCO_2_ gap observations was reported in several studies for patients with and without complications after elective surgery. In the first study of 70 patients undergoing major abdominal surgery, the mean pCO_2_ gap was found to be higher in patients suffering from complications (n = 24, 34%) compared to those patients without complications (7.8 ± 2 mmHg vs. 5.6 ± 2 mmHg, p < 10^−6^) [1]. pCO_2_ gap values were determined at baseline and then hourly until discharge from the PACU. In a second study performed in 115 high-risk surgical patients, the mean pCO_2_ gap was also higher in patients who developed postoperative complications (n = 78, 68%) compared to patients without complications (8.7 ± 2.8 mmHg vs. 5.1 ± 2.6 mmHg, p < 0.001) [16]. The studied population underwent elective major abdominal and vascular surgery and was admitted to the ICU, pCO_2_ gap values were obtained at baseline and then hourly until discharge from the PACU. A third study included 90 patients undergoing major abdominal surgery with scheduled admission to the ICU. The median *intra*operative pCO_2_ gap was higher in patients with complications (n = 39, 43%) compared to patients without complications (6.5 mmHg [5.5–7.3] vs. 5.0 mmHg [3.9–5.8], p < 0.001) [17]. The median *post*operative pCO_2_ gap was higher in patients with complications compared to patients without complications (6.8 mmHg [5.7–8.7] vs. 6.0 mmHg [4.7–7.1], p = 0.03). pCO_2_ gap values were obtained every two hours from baseline to the end of surgery, at ICU admission, and 12 and 24 h after ICU admission.

For comparison purposes, we pooled pCO_2_ gaps at all time points showing similar median pCO_2_ gaps for patients with complications (n = 139, 46%) compared to patients without complications (6.0 mmHg [5.3-7.0] vs. 5.8 mmHg [4.5-7.0], p = 0.078). pCO_2_ gap of patients with complications in our population was low compared to the three previously mentioned studies. Our population consisted of a broader mix of surgical procedures and were not necessarily postoperatively admitted to the ICU. It may be that a relatively healthier population has undergone a relatively less invasive surgery which may partly explain the lack of difference in pCO_2_ gap in our population.

pCO_2_ gap was a poor predictor of major postoperative complications at any of the given time points in our population. Better discrimination was found in the previously mentioned studies as a result of a larger difference in pCO_2_ gap between the groups with and without complications [1, 16, 17].

Pitfalls exist in the interpretation of pCO_2_ gaps. A few mechanisms that affect pCO_2_ gap are the Haldane effect and hyperventilation [18]. Thus, it was suggested that only variations of > 2 mmHg should be considered real variations [19]. pCO_2_ gap obtained at the end of surgery in patients with and without complications did not exceed this threshold.

The GDT group had a significantly higher ScvO_2_ at the end of surgery, which could be related to the interventions as indicated by the treatment algorithm. In addition, higher lactate was found in this group at the end of surgery. This could be caused by the administration of higher volumes of packed red blood cells (not reported here) [10]. With a longer storage time of packed red blood cells lactate concentrations increase [20, 21], although we did not collect this data. For ScvO_2_ as well as lactate, the statistical difference cannot be considered a clinically relevant difference.

We did not find a difference in perioperative pCO_2_ gaps between the GDT and control group in patients undergoing high-risk abdominal surgery. Since cardiac output measurements were absent in the control group of the study, we felt the need to compare adequacy of cardiac output between GDT and standard care by comparing perioperative pCO_2_ gaps as a measure of cardiac output. We therefore hypothesize that both GDT and standard clinical practice led to an adequate cardiac output in our studied population. This may partly help explain why we did not find a difference in major and minor complications and hospital and PACU/ICU length of stay in the original study [10].

Our study has several limitations. First, the control group did not receive additional hemodynamic monitoring and therefore cardiac output was not available. This forced us to use pCO_2_ gap solely as a surrogate for cardiac output without being able to assess the relationship between pCO_2_ gap and cardiac output for this group. Second, it is recommended to use mixed venous pCO_2_ to calculate pCO_2_ gap instead of central venous pCO_2_ [4, 22]. However, since mixed and central venous pCO_2_ have a good agreement, central venous pCO_2_ can be used for this purpose as long as it is not used interchangeably during treatment [22]. Third, we did not obtain data on variables that could have affected pCO_2_ gaps, e.g., hemodilution and body temperature. Fourth, more frequent measurements would have minimized the effect of outliers.

So far, the evidence is inconsistent with the prognostic value of pCO_2_ gaps in a surgical population. Recently, the first trial incorporating pCO_2_ gap in a GDT treatment algorithm has been published [23]. One hundred ASA I/II patients were included and allocated to GDT with ScvO_2_ as a primary target and pCO_2_ gap as a secondary target or to a control group with only arterial blood gas analysis available. No difference was found between the groups in postoperative organ dysfunction (defined by SOFA scores), although the GDT group had a lower length of ICU stay (1.52 ± 0.82 vs. 2.18 ± 1.08 days). Future studies should focus on clarifying the prognostic abilities of pCO_2_ gap by increasing measurement frequencies, including high-risk patients, and recording factors that influence pCO_2_ gaps.

## Conclusion

In conclusion, an association was found between pCO_2_ gap at the end of surgery and major postoperative complications, but pCO_2_ gap was a poor predictor of major postoperative complications at all given time points in our population. Therefore, the use of pCO_2_ gap as a prognostic tool in patients undergoing high-risk abdominal surgery is limited. Moreover, we did not find a difference in of pCO_2_ gap between a GDT and a control group of patients undergoing high risk abdominal surgery, indicating that both patient groups were sufficiently hemodynamically optimized.

## Data Availability

Dataset are not publicly available.
